# Overweight and Obesity Among People Living With HIV on Dolutegravir- and Efavirenz-Based Therapies: A Comparative Cross-Sectional Study

**DOI:** 10.1155/arat/5347620

**Published:** 2024-12-19

**Authors:** Mohammed Jemal, Adane Adugna, Mamaru Getinet, Temesgen Baylie, Nuredin Chura Waritu

**Affiliations:** ^1^Department of Biomedical Science, School of Medicine, Debre Markos University, Debre Markos, Ethiopia; ^2^Department of Medical Laboratory Sciences, College of Health Sciences, Debre Markos University, Debre Markos, Ethiopia; ^3^Department of Biomedical Sciences, School of Medicine, Wolaita Sodo University, Wolaita Sodo, Ethiopia

**Keywords:** dolutegravir, efavirenz, HIV, obesity, overweight

## Abstract

**Background:** Overweight and obesity have arisen as major public health challenges, affecting not just the general population but also people living with human immunodeficiency virus (HIV) (PLWH). Obesity and being overweight are both risk factors for heart disease and other related complications. However, little is known in our setting. As a result, this study was conducted to evaluate the prevalence of overweight and obesity and its associated factors among PLWH on dolutegravir (DTG)- and efavirenz (EFV)-based therapies.

**Methods:** An institution-based comparative cross-sectional study was carried out from June 30, 2021, to August 30, 2021. We purposively recruited 128 participants who have been on DTG (*n* = 64)- and EFV (*n* = 64)-based regimens for ≥ 6 months. Demographic, anthropometric, laboratory, and clinical data were collected using a structured questionnaire. The data were entered into EpiData Version 4.6 and analyzed using SPSS Version 26.0. Multivariable logistic regression was utilized to identify the factors that are associated with being overweight or obese, and the significance level was set at *p* < 0.05.

**Result:** The prevalence of overweight and obesity was 28.1% in the DTG-prescribed participants and 15.6% in the EFV-prescribed participants. Age ≥ 40 years (adjusted odd ratio (AOR) = 3.86; 95% confidence interval (CI): 1.08–13.73; and *p*=0.037), cluster of differentiation 4 (CD4) T-cell counts ≥ 500 cells/mm^3^ (AOR = 2.95; 95% CI: 1.01–8.59; and *p*=0.029), and insufficient physical activity (AOR = 4.6; 95% CI: 1.53–13.84; and *p*=0.007) were predictors of overweight and obesity.

**Conclusion:** Overweight and obesity are not uncommon among PLWH on ART. While the difference was statistically insignificant, the prevalence of overweight and obesity was higher in patients treated with DTG compared with those treated with EFV. Older age, higher CD4 cell counts, and insufficient physical activity were associated with overweight and obesity. As a result, healthcare providers must understand the health implications of obesity and consider incorporating targeted weight control programs into standard HIV treatment.

## 1. Introduction

Human immunodeficiency virus (HIV) infection is typically linked with weight loss or undernutrition [1–3]. However, over the last 20 years, weight gain has been ascribed to the increased availability of highly active antiretroviral therapy (ART), resulting in a significant reduction in the scale of undernutrition among people living with HIV (PLWH) [4]. Nowadays, PLWH on ART can live longer lives, although they are more likely to develop noncommunicable diseases (NCDs) [5]. Among them, metabolic and cardiovascular diseases (CVDs) are the primary causes of death for PLWH in high-income nations [6]. Overweight and obesity are the two risk factors for NCDs such as CVD, diabetes mellitus (DM), dyslipidemia, hypertension, and metabolic syndrome, which complicate HIV management [7, 8]. Furthermore, obesity appears to have a negative impact on immunological recovery following ART initiation [9].

Globally, the rise in overweight and obesity and its effects have increased [10]. This issue is now prevalent in both high-income and low- and middle-income countries [11, 12]. In the United States, 22% of PLWH on ART were overweight and 5% were obese [13]. Sub-Saharan Africa (SSA) suffers from a dual burden of malnutrition, with a high incidence of undernutrition and rising obesity rates, as well as diet-related NCDs. According to studies from low-income countries, overweight and obesity are more prevalent among PLWH (22.1%) [14] and ART-experienced PLWH (34%–35%) [15, 16] than undernutrition (10.0%–26.3%) [14]. In Ethiopia, the pooled prevalence of overweight and obesity among PLWH who are taking ART was found to be 17.85% and 3.58%, respectively [17].

According to the updated HIV prevention, care, and treatment guidelines from the Ethiopian Federal Ministry of Health, the recommended first-line regimen for adults and adolescents is a combination of tenofovir (TDF) + lamivudine (3TC) + dolutegravir (DTG) or efavirenz (EFV) as a once-daily dose [18]. The World Health Organization (WHO) advocates for DTG-based antiretroviral therapy (ART) due to its efficacy, tolerability, and high genetic resistance barrier [19]. Despite its efficacy, various studies have shown that DTG-based treatments are linked to significant weight gain compared with other non-nucleoside reverse transcriptase inhibitors [20–22]. Conversely, recent research has indicated that EFV-based therapies are linked with overweight and obesity as compared to DTG-based therapies [23]. In addition to the ART regimen type, factors such as female gender, African descent, older age, longer duration of HIV and ART, high pretreatment HIV viral load, low baseline cluster of differentiation 4 (CD4)+ count, and low physical activity were also linked with overweight and obesity [24]. Identifying and addressing these risk factors could offer significant benefits for PLWH who are on ART.

Nowadays, in many developing countries, overweight and obesity among PLWH are emerging public health concerns [17, 25]. In Ethiopia, similar to other low- and middle-income countries, the use of DTG is increasing; however, there is limited data on the association between DTG usage and weight gain. Despite this, there is a lack of well-documented information on the prevalence of overweight and obesity among PLWH on DTG- and EFV-based therapies in Ethiopia, specifically in the study area. As a result, the purpose of this study was to determine the prevalence and associated factors of overweight and obesity among PLWH on DTG- and EFV-based ART at Dessie Comprehensive Specialized Hospital, Northeast Ethiopia.

## 2. Methods

### 2.1. Study Area and Period

The study was carried out at the ART clinic of Dessie Comprehensive Specialized Hospital (DCSH) in the South Wollo zone, which is a zone within the Amhara regional state of Ethiopia. The city of Dessie, where the hospital is situated, is located 401 km to the north of Addis Ababa, Ethiopia. As of June 1st, 2021, the hospital provides ART services for more than 6350 HIV/AIDS (6175 adults and 175 pediatric) patients. An institution-based comparative cross-sectional study was conducted at the ART clinic of the DCSH from June 30, 2021, to August 30, 2021.

### 2.2. Population and Eligibility Criteria

The source populations were all PLWH on DTG- and EFV-based therapies at the ART clinic of DCSH. We included all selected ART-naive PLWH aged 18 or older who had been on DTG- or EFV-based therapy (in combination with two NNRTI/NRTI) for more than 6 months. Patients with mental health problems, known DM, hypertension, thyroid disease, pre-existing liver and renal problems, patients on lipid-lowering medications, pregnant and lactating women, and those who refused to participate in the study were excluded.

### 2.3. Sample Size and the Sampling Procedure

The sample size was determined using the G⁣^∗^ Power statistical power analysis Version 3.1 software. This software requires certain inputs, such as selecting an appropriate test family (*t*-test in this case), specifying the alpha (*α*) error probability, power (1 − β error probability), allocation ratio, and effect size. In this study, a two-tailed *t*-test was used to compare two independent sample means with a significance level of *α* = 0.05, a power of 80%, an effect size of *d* = 0.5, and an allocation ratio of 1. Based on these parameters, a total of 128 (64 DTG prescribed and 64 EFV prescribed) participants were determined for the study. The participants were selected using a purposive sampling technique.

### 2.4. Operational Definition

Body mass index (BMI) was calculated using the formula (weight in kg)/(height in meters)^2^. It was categorized into four modalities: undernutrition (< 18.5 kg/m^2^), normal (18.5–24.9 kg/m^2^), overweight (25–29.9 kg/m^2^), and obesity (≥ 30 kg/m^2^). For the risk factor study, BMI was categorized into two modalities: BMI < 25 and BMI ≥ 25 kg/m^2^ [15].

Hypertension was defined as having a systolic blood pressure of ≥ 140 mmHg and/or a diastolic blood pressure of ≥ 90 mmHg [26].

Sufficient physical exercise: for adults, this entails at least 150–300 min of moderate-intensity aerobic physical activity, or at least 75–150 min of vigorous-intensity aerobic physical activity, or an equivalent combination of moderate-intensity and vigorous-intensity activity per week and is, otherwise, insufficient [27].

Low fruit and vegetable intake was defined as consuming < 5 servings of fruit and vegetables per day [28]. For raw green leafy vegetables, 1 serving = one cup; for cooked or chopped vegetables, 1 serving = 1/2 cup; for fruit (banana, orange, etc.), 1 serving = 1 medium-sized piece; for chopped, cooked, and canned fruit, 1 serving = 1/2 cup; and for juice from fruit, 1 serving = 1/2 cup [29].

CD4 cell count ≥ 500 cells/mm^3^ indicates a normal range and a competent immune system, while a CD4 cell count < 500 cells/mm^3^ indicates a compromised immune system [30].

Viral loads ≥ 1000 copies/mL indicate virological failure and high viral loads in the blood whereas viral loads < 1000 copies/mL indicate suppressed viral loads [31].

Abdominal obesity is defined as having a waist circumference > 80 cm for women and > 94 cm for men [32, 33].

### 2.5. Data Collection

#### 2.5.1. Face-to-Face Interview

Data were gathered using a structured questionnaire adapted from the WHO stepwise approach to chronic disease risk factor surveillance [24]. The questionnaire included sociodemographic and behavioral characteristics (age, sex, marital status, residence, educational status, occupation, level of physical activity, and fruit and vegetable intake). The WHO clinical staging, duration of HIV infection, history of opportunistic infection, ART regimen type, duration on ART, CD4 cell count, drug adherence level, and viral load count were obtained from the patient's medical record.

#### 2.5.2. Anthropometric Measurements

Data collectors measured weight using a Tanita scale (to the nearest 0.1 g) and height using a portable stadiometer (to the nearest 0.1 cm), and after that, the BMI of the participant was calculated using the kg/m^2^ formula. Waist circumferences and hip circumferences (to the nearest 0.1 cm) were measured using a flexible inelastic tape.

#### 2.5.3. Blood Pressure Measurements

The data collectors (nurses) at the ART clinic used a standard adult arm cuff and mercury-type sphygmomanometer to assess BP. BP was assessed as the average of the two measurements taken at intervals longer than 5 min after the participants had been sitting for at least 5 or 30 min for those who take hot drinks like coffee.

#### 2.5.4. Biochemical Measurements

A laboratory technologist collected a five (5) mL fasting venous blood sample from each study subject's antecubital vein using a sterile technique. Blood samples were analyzed for fasting blood glucose (FBG) levels and lipid profiles (total cholesterol [TC], high-density lipoprotein cholesterol [HDL-C], low-density lipoprotein cholesterol [LDL-C], and triglycerides [TG]). The analysis of all measurements was carried out using the enzymatic method with a Siemens Dimension EXL 200 System clinical chemistry analyzer.

### 2.6. Data Analysis

The collected information was checked, coded, and entered into EpiData Version 3.1 and analyzed by SPSS Version 26. Descriptive statistics were utilized to ascertain the mean, standard deviation (SD), frequency, and percentage. Categorical variables were shown as numbers and percentages, computed using the chi-square test to identify differences between groups. Meanwhile, continuous variables were displayed as mean ± SD (for normally distributed variables) and median (interquartile range (IQR)) (for skewed variables). The Shapiro–Wilk test was performed to examine whether or not the continuous data distribution was normal. Factors having a *p* value < 0.25 in bivariable analysis were selected for multivariable analysis. Variables with a *p* value of < 0.05 at 95% confidence interval (CI) through multivariable logistic regression were considered statistically significant. Hosmer and Lemeshow's test was used to assess the goodness of fit of the final logistic model, and no problems were found. In addition, the model's fit was checked by a calibration plot, and the points fall on or near the ideal line (45-degree line), so the model is well calibrated (shown in Supporting Figure 1).

### 2.7. Ethical Consideration

This study was conducted in accordance with the Declaration of Helsinki. Ethical clearance with reference number 666/6/2021 was obtained from the Institutional Review Committee of the University of Gondar, and a letter of cooperation was obtained from the DCSH medical director office before the data collection was started. Written informed consent was obtained from the study participants. Privacy and confidentiality of information were kept properly, and their names were not recorded.

## 3. Result

### 3.1. Sociodemographic and Behavioral Characteristics

**Figure 1:**
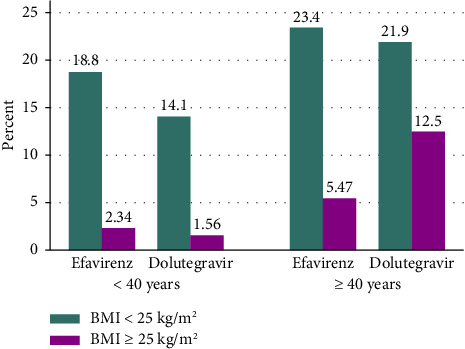
Prevalence of overweight and obesity among PLWH on DTG- and EFV-based ART in relation to age at DCSH.

This study included 128 participants, with an equal number of DTG- and EFV-prescribed participants (*n* = 64 each). More than half of the respondents in both group, 68.8% in the DTG and 57.8% in the EFV, were 40 years of age or older (Figure 1). Between our study groups, a statistically insignificant difference was detected in all of their sociodemographic and behavioral characteristics that are described in Table 1.

### 3.2. Clinical- and Laboratory-Related Parameters

**Figure 2:**
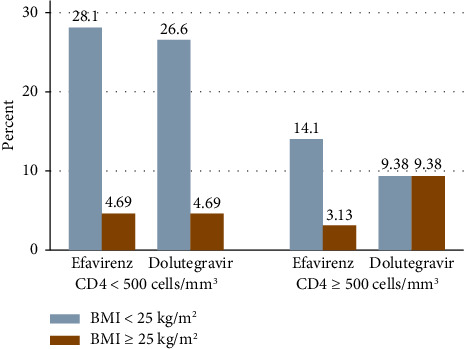
Prevalence of overweight and obesity among PLWH on DTG- and EFV-based ART in relation to CD4 cell count at DCSH.

Nearly two thirds of the study participants, 62.5% of DTG and 65.6% of EFV groups, had CD4 cell counts less than 500 cells/mm^3^ (Figure 2). Out of the total respondents, 92.2% of the DTG-prescribed patients and 87.5% of the EFV-prescribed patients had no history of opportunistic infection in the last 6 months. About 17.2% of DTG-prescribed participants and 9.4% of EFV-prescribed participants had FBG ≥ 110 mg/dL (Table 2).

### 3.3. Prevalence of Overweight and Obesity

**Figure 3:**
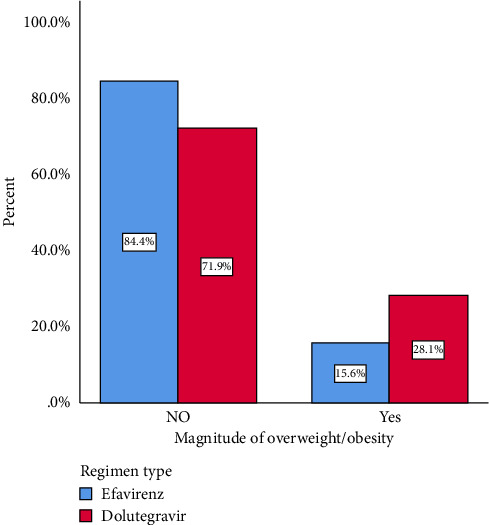
Prevalence of overweight and obesity among PLWH on DTG- and EFV-based ART at DCSH.

The overall prevalence of overweight and obesity among PLWH on ART was 21.9%, with 18.8% classified as overweight and 3.1% as obese (Table 3). According to the regimen type, the prevalence of overweight and obesity was 28.1% in DTG-prescribed participants and 15.6% in EFV-prescribed participants (Figure 3). The prevalence of abdominal obesity was 34.4% in the DTG-prescribed participants and 25% in the EFV-prescribed participants.

### 3.4. Factors Associated With Overweight and Obesity of PLWH on ART

Table 4 shows a binary and multiple logistic regression analysis of the factors associated with overweight and obesity. The multivariable binary logistic regression model included all variables with *p* < 0.25, such as age, gender, education, WHO clinical staging, ART duration, viral load count, CD4 cell count, regimen group, physical activity, TC, HDL-C, and daily fruit and vegetable intake. Age ≥ 40 years (adjusted odd ratio (AOR) = 3.86; 95% CI: 1.08–13.73; and *p*=0.037), CD4 cell counts ≥ 500 cells/mm^3^ (AOR = 2.95; 95% CI: 1.01–8.59; and *p*=0.029), and insufficient physical activity (AOR = 4.6; 95% CI: 1.53–13.84; and *p*=0.007) were significantly associated with overweight and obesity.

## 4. Discussion

There has been a lack of evidence on the possible impact of overweight and obesity among PLWH on DTG- and EFV-based therapies in Africa, notably in Ethiopia. The purpose of this study was to assess the prevalence and predictors of overweight and obesity among PLWH on DTG- and EFV-based therapies.

The overall prevalence of overweight and obesity among PLWH on ART was 21.9%, which is comparable with the estimated prevalence of the general population of Ethiopia (20.4%) [34]. In addition, the findings from Jimma Zone in southern Ethiopia (21%) [35] and Tanzania (25%) [36] were consistent with the results of our study. However, the current finding is slightly higher than study reports from Gamo Zone, southern Ethiopia (13.5%) [23], and Bench Sheko Zone, south-west Ethiopia [37]. On the other hand, the finding of this study is lower than that of studies conducted in Brazil (32.1%) [38], South Africa (overweight: 45.7% and obesity: 23.3%) [39], Botswana (45.4%) [40], Uganda (46%) [41], and Ethiopia (43.4%) [42]. The differences could be attributed to variations in the stages and duration of HIV infection, the type and duration of ART treatment, or differences in the lifestyle and age distribution of the participants studied.

The prevalence of overweight and obesity was 28.1% (overweight: 23.4% and obesity: 4.7%) in the DTG-prescribed participants and 15.6% (overweight: 14.1% and obesity: 1.6%) in the EFV-prescribed participants. We also found that older age, higher CD4 cell counts, and insufficient physical activity were significantly associated with overweight and obesity among PLWH on ART. Our finding is influenced by various confounding factors. First, the EFV-treated patients had a longer duration of HIV/AIDS diagnosis compared with the DTG-treated patients. Second, the duration of ART treatment for EFV-treated patients was almost twice that of DTG-treated patients.

The prevalence of overweight and obesity in our study was comparable in the DTG-based ART (28.33%) but lower in the EFV-based ART (21.94%) when compared with the ADVANCE clinical study conducted in Cameroon [43]. The prevalence of overweight and obesity in the current study was higher in the DTG-prescribed participants and lower in the EFV-prescribed participants as compared with a recent cross-sectional study conducted in Ethiopia, which reported 10.32% and 27.63% among DTG- and EFV-prescribed participants, respectively [23]. There are challenges in comparing our data to previously published studies where the prevalence of overweight and obesity among PLWH on DTG- and EFV-based regimens was studied in noncomparative studies, for instance, a study conducted in Uganda reported that the prevalence of overweight and obesity among DTG-prescribed participants was 29.3%, which is comparable to our findings [44]. In addition, a study conducted in Ethiopia revealed that the prevalence of overweight and obesity among EFV-prescribed participants was 50%, which is higher than the current study [45]. The variation between our study and previous studies could be attributed to differences in sociodemographic characteristics, methodologies (such as sample size, sampling technique, and participants' selection criteria), as well as variation in the duration of HIV infection and ART treatment [46]. For example, a cross-sectional study from Ethiopia was multicentered and had a larger sample size than the current study; in addition, it included alcohol drinkers, smokers, and chat chewers, which were excluded from our study [23]. Likewise, the ADVANCE clinical trials were carried out with a larger sample size than the current study [43].

The exact mechanism in which DTG causes weight gain is not fully understood, but some clinical trials indicated that it might disrupt the central nervous system's ability to regulate appetite, specifically by affecting the melanocortin-4 receptor (MC4R) [47]. This receptor plays a crucial role in regulating caloric intake by modulating leptin signaling and is involved in maintaining energy balance and controlling appetite [48, 49]. Conversely, recent studies have suggested that the weight gain linked to DTG but not EFV might be a result of EFV-related toxicity rather than an unintended effect of DTG. Genetic variations in CYP2B6 that reduce the clearance and metabolism of EFV could lead to higher concentrations of the drug and hinder weight gain [50, 51]. Therefore, the weight gain observed with DTG may indicate a reversal of impaired weight gain caused by such CYP2B6 variations [52].

Our study also attempted to identify risk factors for overweight and obesity among PLWH on ART. Accordingly, participants aged ≥ 40 years were nearly four times more likely to be overweight or obese than those aged < 40. This finding is in line with previous studies that found a link between older age and overweight and obesity [36, 41, 53]. The heightened risk of excess weight and obesity in older adults can be attributed to a reduction in physical activity as well as hormonal changes that occur during aging in both sexes. As a result, decreasing energy expenditure may increase fat accumulation and weight gain [53, 54].

We also observed that CD4 cell count was substantially linked with overweight and obesity. Participants with a CD4 cell count ≥ 500 cells/mm^3^ were 3.6 times more likely to experience overweight and obesity compared with those with a CD4 cell count < 500 cells/mm^3^. Similar results were reported in Ethiopia [35] and USA [55]. This could be due to the fact that a higher initial BMI is linked to larger increases in CD4 cell count, leading to sustained immune status and improved recovery when patients begin antiretroviral drugs [36, 56].

Moreover, the level of physical activity was significantly linked to overweight and obesity. Our study demonstrated that those participants who had insufficient physical activity were 4.54 times more likely to develop overweight and obesity compared with those participants who had recommended physical activity. This result is consistent with findings from South Africa [57], Rwanda [58], and Ethiopia [35]. This association might be explained by the possibility that engaging in physical activity can lower the likelihood of excess weight and may have anti-inflammatory effects for individuals on ART [59]. In addition, increased physical activity increases the total energy expended by individuals. When combined with a reduction in calorie intake, this energy expenditure from physical activity leads to weight loss [60].

### 4.1. Strength and Limitations of the Study

As a strength, to our knowledge, it was among the first studies in Ethiopia to examine the prevalence of overweight and obesity among PLWH on DTG- and EFV-based ART, thus adding to the scant data. However, alongside this strength, there are several limitations to consider. Since it was a cross-sectional study, we cannot establish a causal link between the factors and outcomes being studied. In addition, the study sample size was small, the nonprobability method was used, and the sampling occurred at a single hospital over a defined time period, making it difficult to extrapolate the findings to larger populations. Furthermore, there were baseline differences between the two groups on the duration of HIV/AIDS diagnosis and ART treatment, which could potentially affect the outcome variable. Moreover, the study did not include healthy controls as a comparative group which would have provided better insight into the role of HIV infection and ART.

## 5. Conclusions

In conclusion, overweight and obesity are not uncommon among PLWH on ART. Although the difference was statistically insignificant, the prevalence of overweight and obesity was greater in the DTG-prescribed participants than that in the EFV-prescribed participants. Older, higher CD4 cell counts, and insufficient physical activity were significantly associated with overweight and obesity among PLWH on ART. As a result, healthcare providers must understand the health implications of obesity and consider incorporating targeted weight control programs into standard HIV treatment. There is also a need to promote strategies that encourage physical activity among PLWH on ART. Furthermore, it is important for the Ethiopian national AIDS program to establish guidelines for monitoring and managing overweight and obesity in order to decrease the risk of related health complications. Finally, we recommend that researchers conduct a prospective cohort study with a larger sample size to draw precise conclusions on the prevalence of overweight and obesity among PLWH on DTG- and EFV-based therapies.

## Figures and Tables

**Table 1:** Sociodemographic and behavioral characteristics of PLWH on DTG- and EFV-based ART at DCSH.

Variables	Category	Regimen group		*p* value
		DTG *n* (%)	EFV *n* (%)	
Age	18–39	20 (31.3)	27 (42.2)	0.199
	≥ 40	44 (68.8)	37 (57.8)	

Gender	Male	23 (35.9)	26 (40.6)	0.585
	Female	41 (64.1)	38 (59.4)	

Residence	Urban	42 (65.6)	40 (62.5)	0.713
	Rural	22 (34.4)	24 (37.5)	

Education	Formal education	29 (45.3)	22 (34.4)	0.206
	No formal education	35 (54.7)	42 (65.6)	

Marital status	Married	35 (54.7)	37 (57.8)	0.478
	Unmarried	29 (45.3)	35 (42.2)	

Occupation	Employed	13 (20.3)	8 (12.5)	0.233
	Unemployed	51 (79.7)	56 (87.5)	

Number of servings of FEV on average per day	Low	52 (81.3)	53 (82.8)	0.818
	Recommended	12 (18.8)	11 (17.2)	

Days of FEV intake per week	≤ 4 days	48 (75)	46 (71.9)	0.689
	> 4 days	16 (25)	18 (28.1)	

Level of physical activity	Insufficient	22 (34.4)	27 (42.2)	0.363
	Recommended	42 (65.6)	37 (57.8)	

Abbreviations: DTG, dolutegravir; EFV, efavirenz; FEV, fruit and/or vegetable; N/A, not applicable; No., number; SD, standard deviation.

**Table 2:** Clinical and laboratory-related parameters of PLWH on DTG- and EFV-based ART at DCSH.

Variables	Category	Regimen group		*p* value
		DTG based; *n* (%)	EFV based; *n* (%)	
CD4 count (cells/mm^3^)	≥ 500	24 (37.5)	22 (34.4)	0.713
	< 500	40 (62.5)	42 (65.6)	

Viral load (copies/mL)	Suppressed	59 (92.2)	52 (81.3)	0.068
	Non-suppressed	5 (7.8)	12 (18.8)	

WHO clinical staging	Stage I	48 (75)	42 (65.6)	0.246
	Stage II and above	16 (25)	22 (34.4)	

History of OI in last 6 months	Yes	5 (7.8)	8 (12.5)	0.38
	No	59 (92.2)	56 (87.5)	

Drug adherence level	Good	51 (79.7)	41 (64.1)	0.126
	Fair	5 (7.8)	11 (17.2)	
	Poor	8 (12.5)	12 (18.8)	

Duration of HIV/AIDS (months)	N/A	21.63 ± 8.32	31.69 ± 7.98	0.001⁣^∗^

Duration of ART (months)	N/A	12.0 ± 2.51	22.19 ± 4.45	0.001⁣^∗^

Blood pressure (mmHg)	< 140/90	59 (92.2)	57 (89.1)	0.544
	≥ 140/90	5 (7.8)	7 (10.9)	

Fasting blood glucose (mg/dL)	< 110	53 (82.8)	58 (90.6)	0.193
	≥ 110	11 (17.2)	6 (9.4)	

Total cholesterol (mg/dL)	< 200	47 (73.4)	40 (62.5)	0.185
	≥ 200	17 (26.6)	24 (37.5)	

High-density lipoprotein (mg/dL)^#^	< 40/50	41 (64.1)	25 (39.1)	0.005⁣^∗^
	≥ 40/50	23 (35.9)	39 (60.9)	

Triglyceride (mg/dL)	< 150	37 (57.8)	32 (50.0)	0.375
	≥ 150	27 (42.2)	32 (50.0)	

Low-density lipoprotein (mg/dL)	< 130	51 (79.7)	44 (68.8)	0.157
	≥ 130	13 (20.3)	20 (31.3)	

*Note:* Continuous variables are reported as the mean ± SD and categorical variables are reported as numbers and percentages.

Abbreviations: AIDS, acquired immunodeficiency syndrome; ART, antiretroviral therapy; CD4, cluster of differentiation 4; DTG, dolutegravir; EFV, efavirenz; HIV, human immunodeficiency virus; N/A, not applicable; OI, opportunistic infection; SD, standard deviation; WHO, World Health Organization.

^#^< 40 mg/dL for men and < 50 mg/dL for women.

⁣^∗^Statistically significant at *p* < 0.05.

**Table 3:** Prevalence of overweight and obesity among PLWH on DTG- and EFV-based ART at DCSH.

Status	Regimen group		Total
	DTG-based; *n* (%)	EFV-based; *n* (%)	
Underweight	9 (14.1)	12 (18.8)	21 (16.4)
Normal	37 (57.8)	42 (65.6)	79 (61.7)
Overweight	15 (23.4)	9 (14.1)	24 (18.8)
Obese	3 (4.7)	1 (1.6)	4 (3.1)
Overweight/obese	18 (28.1)	10 (15.6)	28 (21.9)

Abbreviations: ART, antiretroviral therapy; DTG, dolutegravir; EFV, efavirenz.

**Table 4:** Factors associated with overweight and obesity among PLWH on DTG- and EFV-based ART at DCSH.

Variables	Categories	Overweight and obesity		COR (95% CI)	AOR (95% CI)	*p* value
		Yes	No			
Age	< 40 years	5 (10.6)	42 (89.4)	1	1	
	≥ 40 years	23 (28.4)	58 (71.6)	3.33 (1.17–9.48)	3.86 (1.08–13.73)	0.037⁣^∗^

Gender	Male	8 (18.3)	41 (83.7)	1	1	
	Female	20 (25.3)	59 (74.7)	1.74 (0.70–4.32)	1.85 (0.58–5.86)	0.298

WHO clinical staging	Stage I	23 (25.6)	67 (74.4)	2.27 (0.79–6.50)	2.61 (0.71–9.64)	0.150
	Stage II & above	5 (13.2)	33 (86.8)	1	1	

Education	No formal education	6 (11.8)	45 (88.2)	0.33 (0.12–0.89)	0.35 (0.11–1.17)	0.088
	Formal education	22 (28.6)	55 (71.4)	1	1	

ART duration	N/A	15.3 + 4.7	17.6 + 6.6	0.94 (0.87–1.01)	0.92 (0.79–1.09)	0.334

Viral load	Suppressed	22 (19.8)	89 (80.2)	0.45 (0.15–1.36)	0.63 (0.14–2.85)	0.545
	Not suppressed	6 (35.3)	11 (64.7)	1	1	

CD4 cell count	< 500 cells/mm^3^	12 (14.6)	70 (85.4)	1	1	
	≥ 500 cells/mm^3^	16 (34.8)	30 (65.2)	3.11 (1.31–7.37)	2.95 (1.01–8.59)	0.029⁣^∗^

Regimen group	EFV	10 (15.6)	54 (84.4)	1	1	
	DTG	18 (28.1)	46 (71.9)	2.11 (0.89–5.03)	1.36 (0.18–10.14)	0.763

Physical activity	Insufficient	16 (32.7)	33 (67.3)	2.71 (1.15–6.38)	4.6 (1.53–13.84)	0.007 ⁣^∗^
	Recommended	12 (15.2)	67 (84.8)	1	1	

TC	< 200 mg/dL	16 (18.4)	71 (81.6)	1	1	
	≥ 200 mg/dL	12 (29.3)	29 (70.7)	1.84 (0.77–4.36)	1.57 (0.49–5.03)	0.449

HDL-C^#^	< 40/50 mg/dL	17 (25.8)	49 (74.2)	1.61 (0.69–3.76)	1.26 (0.39–4.04)	0.698
	≥ 40/50 mg/dL	11 (17.7)	51 (82.3)	1	1	

Fruit and vegetable intake per day	Insufficient	21 (19.4)	87 (80.6)	0.45 (0.16–1.26)	0.41 (0.11–1.57)	0.194
	Recommended	7 (35.0)	13 (65.0)	1	1	

*Note:* Continuous variables are reported as the mean ± SD and categorical variables are reported as numbers and percentages; Hosmer Lemeshow test *p* value = 0.501.

Abbreviations: AOR, adjusted odd ratio; ART, antiretroviral therapy; CD4, cluster of differentiation 4; CI, confidence interval; COR, crude odd ratio; DTG, dolutegravir; EFV, efavirenz; HDL-C, high density level-cholesterol; N/A, not applicable; TC, total cholesterol; WHO, World Health Organization.

^#^< 40 mg/dL for men and < 50 mg/dL for women.

⁣^∗^Statistically significant at *p* < 0.05.

## Data Availability

The data used to support the findings of this study are available on request from the corresponding author.

## Nomenclature

AOR: Adjusted odd ratio
ART: Antiretroviral therapy
BMI: Body mass index
CD4: Cluster of differentiation 4
CI: Confidence interval
COR: Crude odd ratio
CVD: Cardiovascular disease
DCSH: Dessie comprehensive specialized hospital
DTG: Dolutegravir
EFV: Efavirenz
HDL-C: High-density lipoprotein cholesterol
HIV: Human immunodeficiency virus
SD: Standard deviation
PLWH: People living with HIV
TC: Total cholesterol
WHO: World Health Organization

## Supporting Information

Supporting Figure 1 shows calibration plot for the model's fit.
