# Supplementary figures and images for: Overweight and Obesity Among People Living With HIV on Dolutegravir- and Efavirenz-Based Therapies: A Comparative Cross-Sectional Study
Source: AIDS Res Treat. 2024 Dec 19;2024:5347620. doi: 10.1155/arat/5347620 (PMC11671659; doi:10.1155/arat/5347620)

**SUPPLEMENTARY MATERIAL**


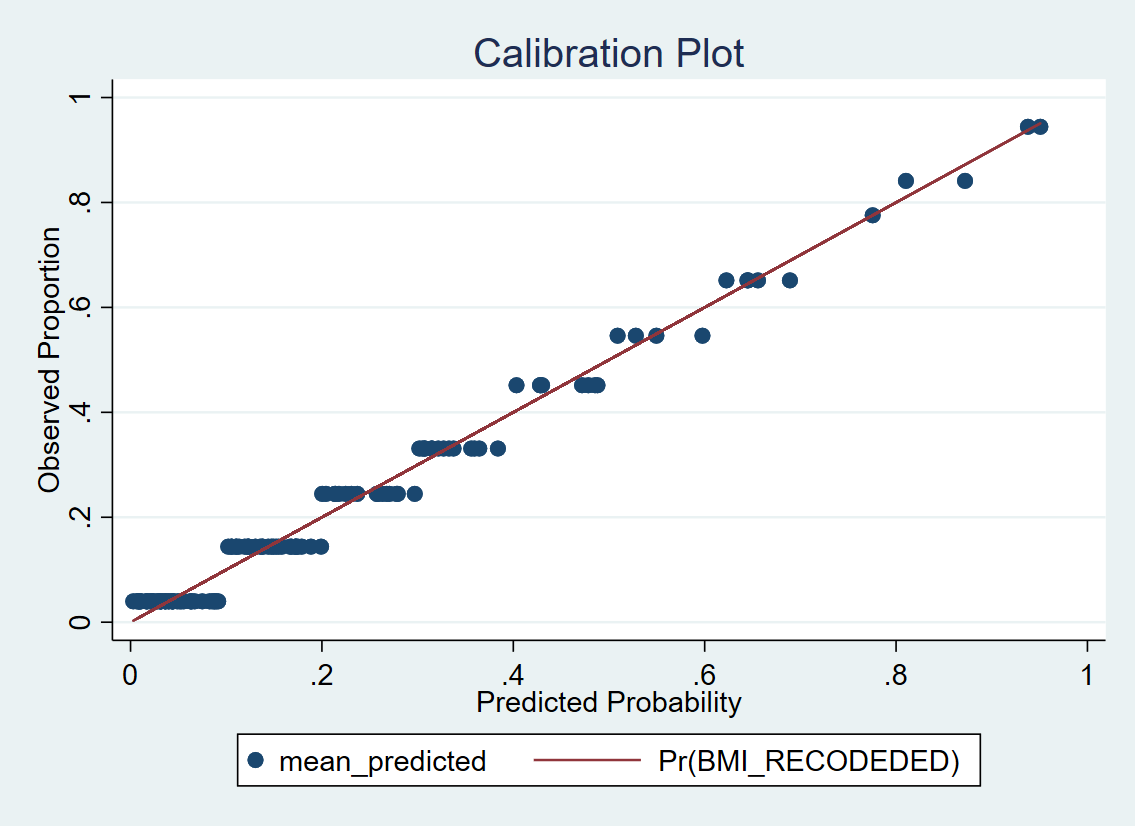


**Supplementary Figure 1** Calibration plot for the model’s fit

Supplement: Supporting Information — Additional supporting information can be found online in the Supporting Information section. [file 5347620.f1.docx]
